# Integration of an iterative factor into a validated HPTLC method for quantification of koumine in the toxic plant *Gelsemium elegans* Benth

**DOI:** 10.3389/fphar.2026.1779290

**Published:** 2026-05-19

**Authors:** Chiew Hoong Ng, Zi Xuan Chim, Chu Shan Tan, Wan Yin Tew, Qiyue Qiu, Idha Kusumawati, Ying Chen, Shuisheng Wu, Mun Fei Yam

**Affiliations:** 1 College of Pharmacy, Fujian University of Traditional Chinese Medicine, Fuzhou, Fujian, China; 2 School of Pharmaceutical Sciences, Universiti Sains Malaysia, Minden, Pulau Pinang, Malaysia; 3 Department of Pharmaceutical Sciences, Faculty of Pharmacy, Universitas Airlangga, Nanizar Zaman Joenoes Building, Surabaya, East Java, Indonesia; 4 NPD3-RG (Natural Products Drug Discovery and Development - Research Group), Faculty of Pharmacy, Universitas Airlangga, Surabaya, East Java, Indonesia

**Keywords:** Gelsemium elegans, HPTLC, iterative factor, koumine, toxic plant

## Abstract

**Background:**

*Gelsemium elegans* (Gardner and Champ.) Benth. (Gelsemiaceae), commonly known as heartbreak grass, is a popular herb to treat a variety of ailments, native to China and Southeast Asia. Its toxicity is attributed to the indole alkaloid it contains, particularly koumine.

**Methods:**

In this research, the iterative factor was integrated into a validated high-performance thin-layer chromatography (HPTLC) method to enhance the precision and reliability of koumine quantification. To establish the validity of the HPTLC assay, precision, repeatability, operating range, robustness, specificity, and recovery analysis were carried out, all of which produced satisfactory results. The amount of koumine for different parts of the *G. elegans* plants across multiple geographical locations was quantified via the validated HPTLC method and derived from the iterative calibration method.

**Results:**

The results obtained from both methods were compared, and the percentage of difference ranged from 0.12% to 3.53%, indicating that there were no significance differences. All samples showed that koumine levels were highest in roots regardless of their origin, but the level of koumine in stems and leaves varied geographically. Samples from Fujian Province showed higher indole alkaloid content in the stem, while the leaf samples from Guangxi Province had higher koumine content.

**Conclusion:**

These findings indicate that the iterative calibration method is suitable for the quantitative analysis of koumine in *G. elegans* Benth and enhances the robustness of HPTLC-based phytochemical standardisation.

## 1 Introduction

*Gelsemium elegans* (Gardner and Champ.) Benth. (Gelsemiaceae) is a poisonous plant distributed across China and Southeast Asia, typically in mountainous areas (Sun et al., 2015). It is also known to the layman in China as *Duan Chang Cao* or *Gou Wen*, (Jin et al., 2014; Wu et al., 2015; Liu et al., 2017). The native hill tribes use the *G. elegans,* which is highly poisonous and potent for suicide via consumption (Rujjanawate et al., 2003). Despite its toxicity, it is still used to treat various ailments, posing a severe public health threat (Xu et al., 2012; Ng et al., 2018). Previous studies discovered that the entire plant of *G. elegans* is poisonous, decreasing in toxicity from root, leaf, flower, fruit, to stem (Hong et al., 1983). Thus, it is in the public interest and concern to precisely identify the chemical identity of certain portions of the plant to minimise public overconsumption due to the significant changes in alkaloid content across the various parts of the plant. This is because the herb still has pharmaceutical properties that are being utilised and produced to manage specific conditions.

HPTLC is the product of systematic achievement, and its fundamental idea is derived from thin-layer chromatography (TLC), which was formerly the favoured approach for herbal analysis prior to the advent of instrumental chromatography (Abhimanyu et al., 2017). HPTLC is currently the standard approach for pharmacopoeia identification and quality assurance of traditional medicines (Fan et al., 2006; Xie et al., 2006; Ankli et al., 2008; Reich and Schibli, 2008; Pudumo et al., 2018).

When combined with appropriate reference materials for inspection, HPTLC provides reliable information for purposes other than basic identification; it concurrently simplifies and enhances quality control for traditional medication production (Ram et al., 2010). The various parts of *G. elegans* were identified using a specific HPTLC approach, yielding an optimised assay and precise identification. The novel concept of “comprehensive HPTLC fingerprinting” is proposed, which entails the conversion of recognised HPTLC fingerprints into peak profiles. The intensities of selected zones are quantitatively compared to the reference material’s corresponding zones (Ram et al., 2010; Frommenwiler et al., 2018).

An iterative factor is an iterative refinement approach that can be applied to improve calibration reliability. This is done by repeatedly adjusting the response factor until the stimulation results align with the experimental data. The simulated values will be compared with the experimental results (Tang and Cui, 2025). This calibration method is fully automated, which requires minimal manual intervention and data preprocessing (Banerjee et al., 2025). To validate the iterative calibration method, the results obtained with and without application of the iterative calibration approach were compared, and the %RSD was calculated (Chen et al., 2021).

Understandably, various investigations have been conducted to isolate and determine the content of koumine in plant extracts using the TLC assay (Zhang et al., 2004; Feng et al., 2013; Sun et al., 2013; Xu et al., 2015). However, there appears to be a dearth of research on developing HPTLC methods for the identification of *G. elegans*. Additionally, previous research has mostly been conducted on TLC plates, which have lower resolution than HPTLC. Thus, this research aims to develop an appropriate HPTLC method for fingerprinting and determining koumine in *G. elegans* in accordance with the US Pharmacopoeia (USP) standard operating procedure (Reich and Schibli, 2008). This can also serve as a framework for developing future HPTLC methods for other plants. The ultimate objective of this study is to characterise the distinctive fingerprint and differentiation of *G. elegans*’ three sections and quantify the amount of koumine contained in the plant using HPTLC.

## 2 Materials and methods

### 2.1 Chemicals and instruments

The reference compound, koumine (>99.84%), was sourced from Chengdu Must Bio-technology Co. Ltd. (Chengdu, China) with voucher number GW34071 deposited in Laboratory of Medica, College of Pharmacy, Fujian University of Traditional Chinese Medicine. Chloroform and ethanol used in this study were purchased from Merck (Darmstadt, Germany), while methanol was purchased from Avantor - J.T.Baker®, USA. Analytical grade chemicals were used in this experiment, and a Milli-Q water purification system was used to purify de-ionised water (Bedford, MA, USA). The microbalance used was the CPA225D (Sartorius, Germany), while the sonicator was GT-2120QTS (GT Sonic Pvt. Ltd, Guangdong, China), and the 0.22-μm PTFE syringe membrane filter was purchased from JinTeng Experiment Equipment Co. Ltd. (Tianjin, China).

### 2.2 Methodology

#### 2.2.1 Preparation of reference solution and internal standard solution

An appropriate amount of koumine was weighed and dissolved in methanol to produce a stock solution of 2 mg/mL. To produce standard working solutions for plotting the calibration curves, the koumine stock solution was diluted into a series of progressively diluted concentrations. The most concentrated stock solution would be 2000 μg/mL, and the lowest concentration was 125 μg/mL.

#### 2.2.2 Sample preparation

A total of 67 *G. elegans* samples including 38 stem samples, 13 root samples, and 16 leaf samples, were provided by Prof Shui-Sheng Wu (Fujian University of Traditional Chinese Medicine, China). These samples were collected from Fujian, Guangxi, and Guangdong provinces within China. After being authenticated, the raw *G. elegans* samples were dried and pulverised into fine powder. 100 mg of the dried powder was subjected to ultrasonication with 1 mL ethanol-water (7:3) for 10 min at 25 °C as an extraction process. The supernatant was then filtered using a 0.45 μm syringe membrane filter to achieve a 100 mg/mL concentration. All solutions were stored at 4 °C until use and sonicated before HPTLC analysis.

#### 2.2.3 Sample application

The sample application was carried out according to the protocols of Teh and Morlock (Teh and Morlock, 2015). Utilising the Automatic TLC Sampler 4 (ATS 4) (CAMAG, Switzerland), the ethanol-water (7:3) extracts (2 µL) were applied as 8 mm bands with a 25 µL syringe onto the HPTLC plate with a spray speed of 150 nL/s. The syringe was rinsed twice with 95% ethanol and twice with the subsequent sample to minimise carryover effects. The first sample was applied onto the HPTLC plate at a distance of 15 mm and 8 mm from the left side and bottom, respectively (Tan et al., 2018). The migration distance was 70 mm from the plate’s lower edge. There are precisely 15 tracks that could be applied when using a 20 × 10 cm plate, out of which six were used for calibration (koumine) and nine for sample (*G. elegans*) application.

#### 2.2.4 Chromatography

Numerous systems of different polarities were investigated to determine the best mobile phase. Ascending development was carried out on 20 × 10 cm HPTLC glass silica gel 60 F254 plates (Merck, Germany) with a mixture of chloroform-methanol-water (30:10:1 v/v/v) as mobile phase in the Automatic Developing Chamber (ADCII) (CAMAG, Switzerland). The twin trough chamber was saturated for 10 min to generate a 33% relative humidity environment before being saturated with mobile phase for 5 min before HPTLC development (Wagner and Bladt, 2008). After development, the plate was dried in the ADCII for 5 min before evaluation.

#### 2.2.5 Plate evaluation

After developing and drying, the plates were viewed under both white and ultraviolet (UV) light at 254nm and 366 nm to analyse koumine qualitatively and quantitatively, and densitometry was used. With the aid of a TLC Visualizer (CAMAG, Switzerland), the plate images were captured under white light and at UV 254 and 366 nm. Using the TLC Scanner 4 (CAMAG, Switzerland), the spectra of the corresponding zones ranged from 200 to 400 nm. A wavelength of 220 nm was adopted for koumine determination because it represents the maximum absorption that ensures optimal sensitivity. Additionally, *in-situ* spectral comparisons confirm a high peak purity at this wavelength. This indicates an absence of matrix interference at this wavelength. The scanning speed was set at 20 mm/s with a macro slit dimension of 5.00 × 0.2 mm. All the gathered data were processed with visionCATS 2.2 (CAMAG, Switzerland). Quantification through peak area was performed using the polynomial calibration function of the respective compounds.

#### 2.2.6 Method validation

The proposed HPTLC method was validated according to the guidelines of the International Conference on Harmonization (ICH) (ICH, 2010). The peaks were analysed using the Rf values’ standard deviation and relative standard deviation. Precision was evaluated by applying calibration standards (as a single replicate per plate, n = 1) to five separate plates on the same day, and reproducibility was determined by repeating the same processes on the samples on four separate days (one plate prepared during the repeatability test, four plates on different days).

Besides that, the working range was determined by applying five different concentrations between 125 and 2000 μg/mL of koumine on a plate. The calculation of the limit of detection (LOD) and the limit of quantification (LOQ) was performed following the formula [LOD = 3.3X (SE/S) and LOQ = 10X (SE/S)], where SE stands for the standard error of low-level concentration, and S stands for the slope of the linear range of the calibration curve (Attimarad et al., 2011; Abhimanyu et al., 2017).

The robustness of research is defined as the method’s capacity to accept parameter changes without significantly affecting the obtained data. This can be determined by evaluating the volume of the mobile phase, equilibration time between the mobile phase and the stationary phase, and the dosage speed of sample application. The mobile phase volume was performed in 5, 10, and 15 mL, the equilibration time of the mobile phase and the stationary phase was performed at 10, 20, and 30 min, and the dosage speed of sample application was 100, 150, and 200 nL/s to observe if there are any significant differences between the variables. The comparative standard conditions are 10 mL of developing solvent at 20 min equilibration time with a spraying speed of 100 nL/s.

A specificity test was also conducted by comparing the koumine bands in the corresponding samples in terms of the *Rf* value and UV spectra with standard solutions (Tan et al., 2018). The recovery studies with *G. elegans* extracts were performed to study the method’s accuracy. In the study, 500 µL *G. elegans* extract was independently prepared and was spiked with 500 µL standard solution. They were applied as a single replicate per plate (n = 1).

#### 2.2.7 Iterative calibration approach

After the validation of the HPTLC method, a new calibration concept was applied for the sample analysis portion. First, koumine (0.0625, 0.125, 0.25, 0.5, and 1 mg/mL) (calibration curve) was applied separately in HPTLC plates alongside the *G. elegans* samples. Then, the concentration of koumine in each sample was determined from the calibration curve generated from the same plate. After that, the mean calibration curve was calculated from the HPTLC analysis, giving the equation of y = 8.08E-04x^2^ + 0.0406x − 0.0188 with an R^2^ of 0.998. This equation was used to calculate the koumine content of the sample on each plate.

The amount of koumine in the samples was determined by using a mean calibration curve and multiplying by an iterative factor, which is calculated from the sample plate. This factor is the ratio of the peak area of Koumine 0.25 mg/mL obtained from the mean calibration curve to the peak area of Koumine 0.25 mg/mL from the data obtained. The iterative calibration analysis method was validated via comparison of the outcomes with and without an iterative calibration approach. The percentage of difference between the concentration obtained from the mean calibration curve and the concentration from the calibration curve of a specific plate was calculated from this iterative calibration analysis for validation purposes.

$$\text{Iterative factor}=\frac{\text{Peak}\;\text{area}\;\text{of}\;\text{koumine}\;0.25\text{mg}/\text{mL}\;\text{from}\;\text{mean}\;\text{calibration}\;\text{curve}}{\text{Peak}\;\text{area}\;\text{of}\;\text{koumine}\;0.25\text{mg}/\text{mL}\;\text{from}\;\text{result}\;\text{obtained}}$$

The reliance on a single-point iterative factor can introduce uncertainty. This issue was addressed by propagating the standard uncertainty of the iterative factor into the final concentration estimates. The relative standard deviation obtained from the intra-day precision of the 0.25 mg/mL standard was combined with the standard error of the mean calibration curve using the root-sum-square method. Therefore, the expanded uncertainty for the reported koumine concentrations can be calculated.

To evaluate the analytical equivalence between the standard plate-specific calibration and the proposed iterative approach for koumine quantification, a Bland-Altman analysis was performed. This analysis can evaluate the systematic bias and define the limits of agreement between the two quantitative approaches. Before conducting this analysis, an *a priori* acceptable equivalence margin was established according to AOAC Guidelines, which is an acceptable margin of ±5% was defined for alkaloid quantification in *G. elegans* (AOAC-International, 2002). This 5% relative margin corresponds to an absolute acceptable difference of approximately ±0.15 μg/mg at the lower bound of quantification.

## 3 Results and discussion

### 3.1 Fingerprint of Gelsemium elegans

**FIGURE 1:**
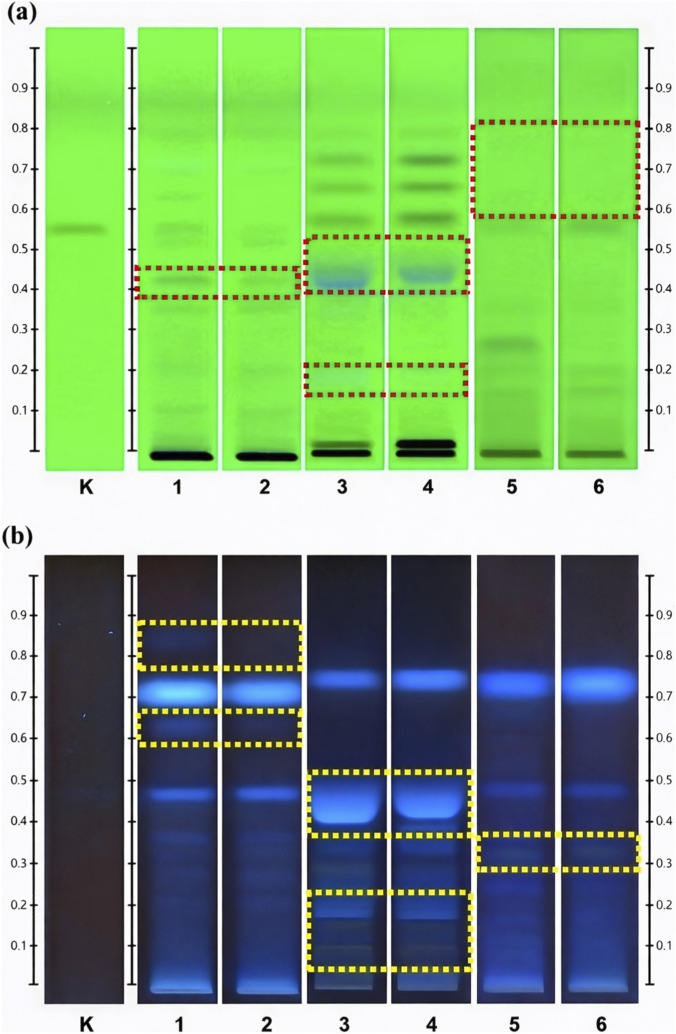
The HPTLC plate observed under: **(a)** UV 254 nm; and **(b)** UV 366 nm for koumine (Track K), stem from Fujian Province (Track 1), stem from Guangxi Province (Track 2), root from Fujian Province (Track 3), root from Guangxi Province (Track 4), leaf from Fujian Province (Track 5), and leaf from Guangxi Province (Track 6) with retardation factor (Rf) scale at both sides.

In Figures 1a,b, the HPTLC fingerprint representing different parts of *G. elegans* was visualised at UV 254 and 366 nm. The reference compound, koumine (track K), has its fingerprints that can be seen at UV 254 nm with an *Rf* value of 0.58 ± 0.02. Due to the vast number of samples, head-to-head comparisons of differences across samples were not possible. Thus, to better understand the main highlights of HPTLC fingerprinting comparisons between different parts of *G. elegans*, one sample from each province and various parts of *G. elegans* were selected for comparison. Figure 1 illustrates a side-by-side comparison of samples from different parts and provinces.

As shown in Figure 1, tracks 1 and 2 reflect the fingerprints of *G. elegans* stems from Fujian and Guangxi provinces, respectively. Under UV light of 254 nm, the stem fingerprints developed a band at Rf 0.40. However, UV light at 366 nm displays a bright pink band at Rf 0.62 and a light blue band at Rf 0.82. These bands are unique to the fingerprints of *G. elegans* stem samples and are not present in the analysis for other parts of the plant (root and leaf). This enables us to confidently conclude that these specific bands might be utilised to differentiate stem samples from root and leaf samples.

Numerous detection modes were used to facilitate the identification of the plant’s roots on tracks 3 (Fujian) and 4 (Guangxi), depending on different zones between the application position and the koumine zone (Rf 0.58, track K). The root fingerprint displayed blue bands between Rf 0.18 and 0.40 at UV 254 nm, but a yellow band around Rf 0.80 at UV 366 nm, two blue bands between Rf 0.15 and 0.20, and a broad blue band around Rf 0.40 and 0.50 at UV 366 nm. These bands are unique to root samples and represent a distinguishing feature not observed in stem or leaf sample analysis.

The leaves and their samples overall yielded a relatively weak fingerprint. However, certain characteristic bands remain and can uniquely identify the samples. These fingerprints are displayed in tracks 5 and 6 for samples collected in Fujian and Guangxi provinces. As seen in Figure 1, it does not exhibit any bands between Rf 0.60 and 0.90 at UV 254 nm, in contrast to the stem and root samples, which display at least four bands within this range. The lack of these peaks can also identify the leaf samples as a differentiating trait. Under UV 366 nm, the leaf fingerprint yielded an intriguing result. Extra bands were seen, and two red patches were between Rf 0.58 and 0.70 and Rf 0.78 and 1.0. These red patches were not visible in the prior root or stem samples. Additionally, a faint yellow band was discovered around Rf 0.32 in the leaf fingerprint, separating the leaf from the stem and root.

However, there were insignificant differences when comparing samples from the different provinces. Unlike the fingerprint of UPLC, the HPTLC fingerprints from the two other provinces showed almost identical fingerprints, and we cannot identify apparent distinguishing features. This might be owing to the HPTLC model’s lack of sensitivity, which is unlikely to identify minor chemical variations between samples from the two provinces.

By converting the visual plate data to objective mathematical modeling, the geographical or species differences can be distinguished. Hence, integration of these comprehensive peak profiles with chemometric analysis allows objective, large-scale geographical differentiation (Bārzdiņa et al., 2022).

### 3.2 Method validation

#### 3.2.1 Precision

Precision within a day was evaluated by utilizing calibration standards on five different plates. Table 1 illustrates the intra-day precision results. The difference in average Rf values between the two selected zones varies by < 0.02 from plate to plate. The RSD% is less than 3.7736.

**TABLE 1:** Relative standard deviation expressed in percentages of known concentrations of koumine in intra-day precision and inter-day reproducibility tests.

Concentration (µg/mL)	Intra-day precision RSD (%)	Inter-day reproducibility RSD (%)
125	3.6082	3.8781
250	3.7736	2.7730
500	2.9773	2.5772
1,000	1.7697	1.5906
2,000	1.2907	1.9726

#### 3.2.2 Reproducibility

The protocol’s reproducibility was evaluated by repeating the analysis on four separate days. The inter-day reproducibility data are provided in Table 1, with a difference of <0.05 between the average values of plates on different days. The RSD% is less than 3.8781.

#### 3.2.3 Working range, limit of detection (LOD) and limit of quantification (LOQ)

**FIGURE 2:**
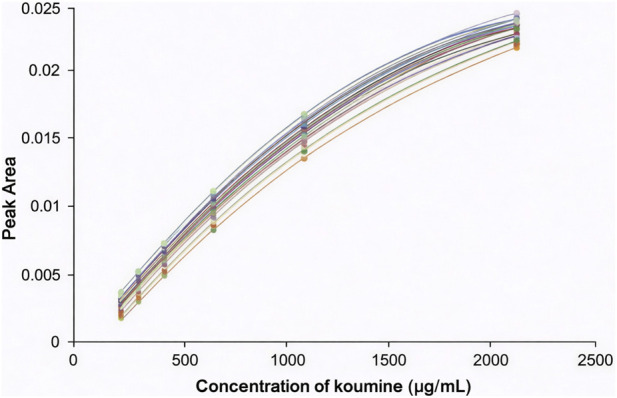
Calibration curves were obtained from the graph of peak area versus concentration of koumine (µg/mL).

A calibration curve based on a second-order polynomial function was formed by applying five concentrations of koumine solution ranging from 125 to 2000 μg/mL on a plate. The calibration is polynomial regression-based due to the higher calibration range. The second-order model was chosen to appropriately justify the non-linear densitometric response and inherent heteroscedasticity that are commonly observed at higher concentrations in HPTLC. The 67 samples were quantified using 28 plates, and the calibration curve for all plates is presented in Figure 2. The equations and correlation of determination (R^2^) for the 28 calibration curves are reported in Table 2. All calibration curves have an R^2^ greater than 0.9995.

**TABLE 2:** Equation and R^2^ for the calibration curves of the 28 plates.

Plate	Equation	R^2^
1	y = -3E-09x^2^ + 2E-05x + 0.0005	0.9999
2	y = -3E-09x^2^ + 2E-05x + 0.0003	0.9999
3	y = -3E-09x^2^ + 2E-05x + 0.0002	1
4	y = -3E-09x^2^ + 2E-05x + 8E-05	0.9999
5	y = -3E-09x^2^ + 2E-05x + 0.0002	0.9999
6	y = -3E-09x^2^ + 2E-05x + 0.0002	1
7	y = -3E-09x^2^ + 2E-05x + 3E-05	1
8	y = -3E-09x^2^ + 2E-05x - 0.0004	0.9999
9	y = -3E-09x^2^ + 2E-05x + 0.0004	0.9999
10	y = -4E-09x^2^ + 2E-05x + 0.0004	1
11	y = -4E-09x^2^ + 2E-05x + 0.0004	0.9999
12	y = -4E-09x^2^ + 2E-05x + 0.0002	0.9998
13	y = -3E-09x^2^ + 2E-05x - 2E-05	0.9995
14	y = -3E-09x^2^ + 2E-05x + 8E-05	0.9996
15	y = -3E-09x^2^ + 2E-05x - 0.0008	0.9996
16	y = -3E-09x^2^ + 2E-05x + 0.0004	0.9998
17	y = -3E-09x^2^ + 2E-05x + 0.0002	0.9997
18	y = -3E-09x^2^ + 2E-05x + 0.0015	0.9998
19	y = -2E-09x^2^ + 1E-05x + 0.0013	0.9997
20	y = -3E-09x^2^ + 2E-05x + 0.0009	0.9998
21	y = -3E-09x^2^ + 2E-05x + 0.0003	0.9998
22	y = -3E-09x^2^ + 2E-05x - 0.001	0.9997
23	y = -4E-09x^2^ + 2E-05x + 0.0004	0.9997
24	y = -4E-09x^2^ + 2E-05x + 0.0007	0.9999
25	y = -2E-09x^2^ + 2E-05x - 0.0004	0.9999
26	y = -3E-09x^2^ + 2E-05x + 0.0003	0.9998
27	y = -4E-09x^2^ + 2E-05x - 0.0003	0.9996
28	y = -4E-09x^2^ + 2E-05x + 0.0009	1

Following the determination of the LOD and LOQ for each plate, they were both standardised by determining the mean value, resulting in a mean LOD of 30.6595 μg/mL and a mean LOQ of 92.9075 μg/mL.

#### 3.2.4 Robustness

##### 3.2.4.1 Volume of developing solvent

The same analysis was performed using three different volumes of the mobile phase: 5, 10, and 15 mL. To verify the study’s robustness, an acceptable criterion for variation in Rf values due to developing solvent volume variation would be less than 0.05. No significant variations were found when the plates were developed in the three different volumes of the mobile phase (Table 3).

**TABLE 3:** Changes in the fingerprint analysis of the tested fractions as function of volume of developing solvent.

Samples	5 mL	10 mL	15 mL	Mean	RSD %
Koumine	0.570	0.580	0.590	0.580	1.7241
Stem	0.570	0.580	0.580	0.577	1.0006
Leaf	0.580	0.590	0.590	0.587	0.9836

##### 3.2.4.2 Equilibration time

When the equilibration time for the mobile phase and the stationary phase is changed to 10, 20, or 30 min, the acceptance criteria for the difference in Rf values as a function of equilibrium time is <0.05. The separation was not affected by equilibration time (Table 4).

**TABLE 4:** Changes in the fingerprint analysis of the tested fractions as function of equilibration time of gas phase and stationary phase.

Samples	10 min	20 min	30 min	Mean	RSD %
Koumine	0.550	0.580	0.590	0.573	3.6308
Stem	0.550	0.580	0.600	0.577	4.3641
Leaf	0.560	0.580	0.600	0.580	3.4483
Root	0.550	0.580	0.610	0.580	5.1724

##### 3.2.4.3 Dosage speed of sample applicator

The dosage speed of the sample applicator is the other variable that could potentially affect the results of the assay. As a result, the study’s robustness was evaluated by adjusting the dosage speed to administer the sample at 100, 150, or 200 nL/s. Similar to the other variables, the acceptance criterion for the difference of *Rf* values as a function of dosage speed would be less than 0.05. Increased dosage application speed had no discernible effect (Table 5).

**TABLE 5:** Changes in the fingerprint analysis of the tested fractions as function of dosage speed of sample applicator.

Samples	100 nL/s	150 nL/s	200 nL/s	Mean	RSD (%)
Koumine	0.58	0.58	0.57	0.577	1.0012
Stem	0.59	0.58	0.58	0.583	0.9897
Leaf	0.59	0.58	0.57	0.580	1.7241
Root	0.59	0.57	0.57	0.577	2.0024

#### 3.2.5 Specificity

**FIGURE 3:**
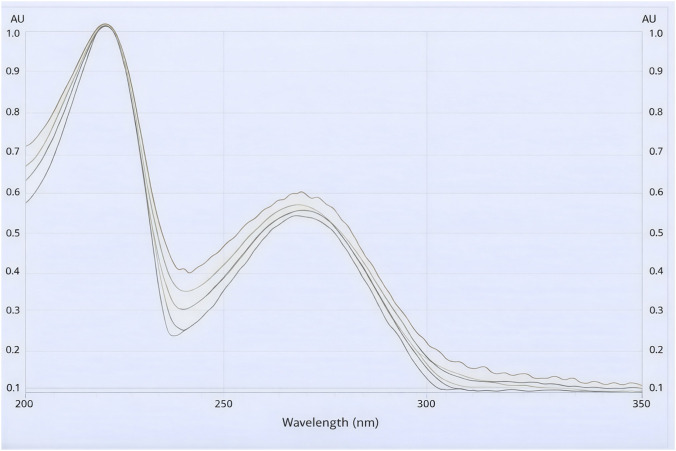
UV spectrum of koumine and koumine band in the stem, root, and leaf of *Gelsemium elegans.*

The maximum absorption wavelength of koumine was confirmed to be 220 nm, as illustrated in Figure 3. All UV spectra are identical (correlation coefficient, *R* > 0.99), showing that this method is specific for koumine. The correlation of determination for every sample is shown in Table 6.

**TABLE 6:** The mean amount of koumine in *Gelsemium elegans* sample and their respective RSD and correlation coefficient with pure koumine.

Sample	Mean (ug/mg)	RSD (%)	Correlation coefficient (specificity)
GW001-S	2.473 ± 0.097	3.92	0.9968
GW002-S	1.273 ± 0.071	5.58	0.9972
GW003-S	1.308 ± 0.068	5.20	0.996
GW004-S	1.155 ± 0.046	3.98	0.9902
GW005-S	--	--	--
GW006-S	4.220 ± 0.216	5.12	0.9981
GW007-S	4.399 ± 0.179	4.07	0.9995
GW008-S	2.843 ± 0.122	4.29	0.999
GW009-S	--	--	--
GW010-S	5.629 ± 0.218	3.87	0.9975
GW011-S	2.708 ± 0.106	3.91	0.9973
GW012-S	7.454 ± 0.218	3.77	0.9979
GW013-S	3.031 ± 0.118	3.89	0.9964
GW014-S	1.500 ± 0.057	3.80	0.9941
GW015-S	2.583 ± 0.099	3.83	0.9975
GW016-S	1.223 ± 0.063	5.15	--
GW017-S	4.000 ± 0.194	4.85	0.997
GW018-S	4.533 ± 0.191	4.21	0.997
GW019-S	4.376 ± 0.165	3.77	0.9982
GW020-S	4.920 ± 0.196	3.98	0.9973
GW021-S	4.234 ± 0.164	3.87	0.9966
GW022-S	--	--	--
GW023-S	3.299 ± 0.172	5.21	0.9977
GW024-S	1.147 ± 0.044	3.84	--
GW025-S	1.637 ± 0.071	4.34	0.9947
GW026-S	2.349 ± 0.098	4.17	0.9913
GW027-S	1.466 ± 0.095	6.48	0.9985
GW028-S	--	--	--
GW029-S	--	--	--
GW030-S	1.645 ± 0.070	4.26	0.9916
GW031-S	1.130 ± 0.046	4.07	0.9976
GW032-S	1.518 ± 0.059	3.82	0.9903
GW033-S	1.879 ± 0.088	4.58	0.998
GW034-S	1.539 ± 0.066	4.29	0.9992
GW035-S	1.211 ± 0.079	6.61	--
GW036-S	2.008 ± 0.088	4.33	0.9989
GW037-S	--	--	--
GW038-S	1.686 ± 0.116	6.88	0.9961
GW039-R	7.000 ± 0.370	5.29	0.9907
GW040-R	5.159 ± 0.212	4.11	0.993
GW041-R	9.000 ± 0.352	3.80	0.9989
GW042-R	3.717 ± 0.217	5.87	0.9972
GW043-R	6.067 ± 0.234	3.79	0.9965
GW044-R	1.322 ± 0.052	3.86	0.9982
GW045-R	6.356 ± 0.261	4.06	0.9977
GW046-R	7.561 ± 0.329	4.32	0.9957
GW047-R	3.294 ± 0.124	3.76	0.9972
GW048-R	7.418 ± 0.281	3.77	0.9977
GW049-R	--	--	--
GW050-R	4.489 ± 0.174	3.79	0.9936
GW051-R	5.696 ± 0.222	3.79	0.9911
GW052-L	--	--	--
GW053-L	3.036 ± 0.118	3.79	0.9905
GW054-L	--	--	--
GW055-L	--	--	--
GW056-L	1.945 ± 0.128	6.58	--
GW057-L	--	--	--
GW058-L	--	--	--
GW059-L	4.281 ± 0.181	4.04	0.9927
GW060-L	1.609 ± 0.074	4.60	0.9902
GW061-L	--	--	--
GW062-L	1.032 ± 0.047	4.55	--
GW063-L	2.255 ± 0.108	4.74	0.9921
GW064-L	--	--	--
GW065-L	1.578 ± 0.072	4.56	0.9907
GW066-L	3.044 ± 0.154	5.03	0.9903
GW067-L	1.592 ± 0.086	5.34	0.9918

“-” denotes detection under the threshold of LOQ.

#### 3.2.6 Recovery studies (accuracy)

The accuracy was expressed as a percentage value, and it represents the percentage of the mean value to the computed value, as shown in Table 7 (Abhimanyu et al., 2017).

**TABLE 7:** Results of recovery tests for koumine.

Sample	Original content (µg/mL)	Added content (µg/mL)	Detected content (µg/mL)	Recovery %	RSD %
Stem	247.30	250.00	500.68 ± 19.46	109.49	3.89
Leaf	228.10	250.00	456.49 ± 18.31	95.48	4.01
Root	498.80	500.00	987.40 ± 37.29	98.86	3.78

#### 3.2.7 Iterative calibration

The single reference concentration for the derivation of iterative factor was selected to be 0.25 mg/mL. The selection was established via a sensitivity analysis of different reference levels which are 0.0625, 0.125, 0.25, 0.5 and 1.0 mg/mL. Each concentration for evaluated for their robustness by calculating the percentage of difference between the observed and calculated results across a representative subset of 10 independent plates. According to Table 8, the iterative factor derived from the concentration of 0.25 mg/mL yielded the lowest percentage of difference which is 0.68%. This concentration is at the optimal threshold in the working range because it has sufficient signal-to-noise characteristics to overcome the additive baseline fluctuations at lower limits. This concentration was also well below the non-linear upper boundaries of the densitometric response. Hence, 0.25 mg/mL is the concentration that highly represents the overall method performance and it provides the most robust scaling adjustment.

**TABLE 8:** Sensitivity analysis evaluating the percentage difference between calculated and observed results when utilizing different reference concentrations for the iterative factor.

Plate/Sample	0.0625 mg/mL	0.125 mg/mL	0.25 mg/mL	0.5 mg/mL	1 mg/mL
Plate 1	−31.75%	9.42%	−3.74%	1.52%	2.41%
Plate 10	−28.83%	7.39%	−0.21%	2.95%	14.46%
Plate 11	−39.84%	3.41%	−3.69%	2.43%	13.07%
Plate 12	75.34%	4.80%	−0.09%	17.54%	5.72%
Plate 21	42.06%	14.68%	1.36%	−1.54%	9.94%
Plate 23	−20.93%	2.39%	5.78%	4.64%	5.29%
Plate 25	11.72%	5.36%	4.60%	6.74%	27.66%
Plate 26	−25.30%	−8.76%	−0.07%	−0.91%	6.69%
Plate 27	−14.85%	−13.19%	1.00%	−1.04%	4.51%
Plate 28	0.08%	−10.69%	1.87%	−0.51%	5.39%
**Average**	−3.23%	1.48%	0.68%	3.18%	9.51%

Bold values indicate the lowest percentage difference and optimal concentration.

By using the iterative calibration method with iterative factor derived from 0.25 mg/mL, the average percentage of difference between the calculated and the observed concentration of the koumine samples ranged from 0.12% to 3.53%. The finding suggests that the iterative calibration method can be used to estimate the concentration of koumine in each sample from the peak area alone.

The developed method was validated by the value of variance, which is 1.69%, which is less than 2%. Variance measures the difference between numbers in a data set to calculate the degree of dispersion of data around the sample’s mean. The yield value is highly restricted (less than 2%) which indicates that applying the mean calibration curve does not introduce unpredictable dispersion into the calculated concentrations when compared to the plate-specific calibration.

The result is also analysed using the dependent sample t-test. By using this method, we can statistically compare the proposed method with an existing or reference method. In this test, each object or entity is measured twice, resulting in two sets of observations (Hasija, 2023).

From the results of the paired t-test, we obtained a value of P > 0.05 (Table 9). This indicates that the null hypothesis cannot be rejected. Thus, there is no significant difference between the data obtained from the HPTLC plate and the data obtained from the iterative calibration approach.

**TABLE 9:** Results of paired samples test.

Pair	Sig. (2-Tailed)	t-value	Correlation
Plate calibration - mean calibration	0.506	0.668	0.987

However, the inherent statistical dependence in the comparison using paired t-test and correlation analysis should be acknowledged. It is because the iterative factor is directly derived from the 0.25 mg/mL reference standard of the plate, which is also the same data point used to generate the traditional plate-specific calibration curve. The same data point used can introduce circularity into the model and suppresses the variance of the differences. Thus, the apparent statistical agreement in the paired t-test might be inflated. To diminish this dependence and validate this method, the recovery studies result as shown in Table 7 serves as an important external validation. The spiked samples used in this recovery analysis represent independent reference measurements that were not involved in the derivation of the iterative factor. As the result showed that the iterative calibration approach successfully quantified these independently spiked samples, it indicated that the normalization strategy has high analytical accuracy.

In addition, we also get a t-value of 0.668, which is considered small (Table 9). It means that the observed difference is small compared to the variation in the data. A t-value that is positive shows that the value obtained from the HPTLC plate is slightly higher than the value obtained from the mean calibration curve. However, the difference is small and well within the random noise.

The value of correlation is 0.987 (Table 9). Correlation measures the monotonic association between 2 variables. According to the conventional approach to interpreting a correlation coefficient, a correlation coefficient that lies in the range of 0.90–1.00 is interpreted as a very strong correlation (Schober et al., 2018). The correlation value is 0.987, which lies in the range of 0.90–1.00, which means the paired data has a very strong correlation. Therefore, the paired data are linearly related to each other and vary closely together.

Both results obtained from calculating variance and the paired t-test show that the data obtained from the HPTLC plate and the data obtained from the mean calibration curve vary in a non-significant range. Therefore, it proves that the concentration of a specific substance can be determined by using a mean calibration curve. There is no need to generate a new calibration curve for every plate.

Based on the Bland-Altman plot (Figure 5), the differences between the values obtained from the HPTLC plate-specific calibration and those derived from the mean calibration curve were plotted against their respective means. The analysis had shown that the mean difference between the two methods is 0. This result indicates an absolute absence of systematic error or proportional bias when the standard calibration is substituted with the iterative factor approach. Furthermore, the 95% Limits of Agreement span from a lower limit of −0.07 to an upper limit of +0.06.

Moreover, the majority of the paired data points are distributed closely around the zero-bias line and fall strictly within the 95% LoA. There are only a few outliers because the calculated 95% limits of agreement (−0.07 to +0.06) are narrow and fall well within our *a priori* defined acceptable equivalence margins. These findings indicate that the two methods are deemed analytically equivalent. Applying the iterative factor does not compromise the quantitative integrity of the assay. Therefore, this method can be reliably used in plate-specific calibration curves for the quantification of koumine.

A ratio plot that compares the observed response to the mean response across the calibration range was generated for all plates to validate the assumption that inter-plate variability is primarily multiplicative. Based on Figure 6, the analysis revealed a mixed-error profile that varies depending on the concentration range. The ratio exhibited wider variance and sloped trends at the lower end of the calibration curve. This indicates a significant contribution of additive error that is likely caused by the inherent baseline noise and stationary phase variations. At concentrations of 0.25 mg/mL and above, the ratio trend lines were stabilized with improved parallelism. This confirms that inter-plate variability is predominantly multiplicative in the primary working range. Hence, deriving the iterative factor from the 0.25 mg/mL standard is analytically justified. This is because the iterative factor represents the threshold where multiplicative scaling accurately corrects for plate-to-plate response variations without being overly skewed by additive baseline noise.

To ensure that the koumine levels accurately reflect the analytical adjustment, a formal uncertainty propagation was conducted. By applying the standard error propagation rules for multiplicative models, the three sources of variance were included in the total measurement uncertainty. They were the regression uncertainty of the mean calibration model, the repeatability uncertainty of the sample peak areas and the relative uncertainty of the iterative factor. The iterative factor was directly derived from the reference response. Thus, the relative uncertainty of the iterative factor was conservatively approximated as 3.77% according to the intra-day precision variance of the calibration standards. By using the root-sum-square method, the variances were combined into the final concentration estimates.

To assess the practical impact, the expanded uncertainty was compared with the conventional plate-specific calibration and the proposed iterative approach for low, mid and high concentration samples. After applying the iterative factor, the relative expanded uncertainty ranged from ∼4.5% to ∼8.8% on average. The intention of the applying this HPTLC protocol is for routine quality control and authentication of botanical materials instead of strict regulatory release testing. Therefore, a relative expanded uncertainty of not more than 10% represents an acceptable analytical performance. It successfully maintains fit-for-purpose reliability and significantly reduces the calibration standard load on each plate.

### 3.3 Quantification of *Gelsemium elegans*

**FIGURE 4:**
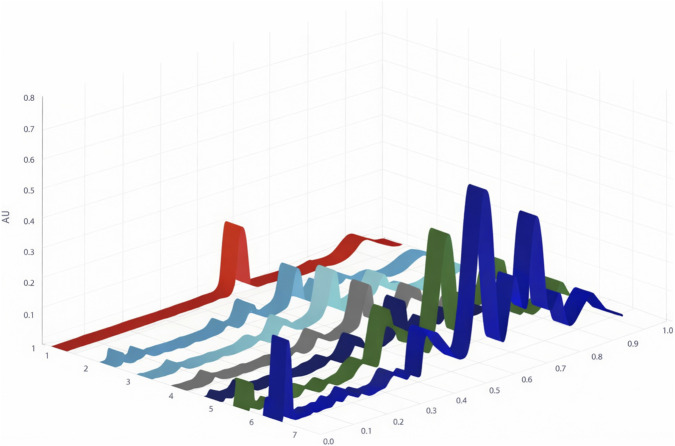
HPTLC chromatograms of koumine, stem from Fujian Province, stem from Guangxi Province, root from Fujian Province, root from Guangxi Province, leaf from Fujian Province, and leaf from Guangxi Province with retardation factor (Rf) scale at both sides. Track 1: koumine, Track 2: stem from Fujian Province, Track 3: stem from Guangxi Province, Track 4: root from Fujian Province, Track 5: root from Guangxi Province, Track 6: leaf from Fujian Province, Track 7: leaf from Guangxi Province.

The calibration was determined by comparing the concentration of the standard compounds to their area under the curve. The calibration curve of koumine is shown in Figure 2, and the chromatograms of the selected samples are shown in Figure 4. The quantity of koumine in samples is determined by calculating the area under the curve of the bands whose Rf values correlate with koumine. The determined koumine content in *G. elegans* is summarised in Table 6. When the quantities of the tested compounds were not discovered in samples or were less than the quantification limit, the values were judged to be zero.

**FIGURE 5:**
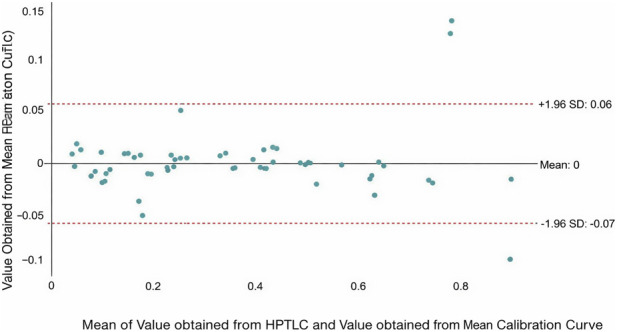
Bland-Altman Plot of Value obtained from HPTLC and Value obtained from Mean Calibration Curve.

**FIGURE 6:**
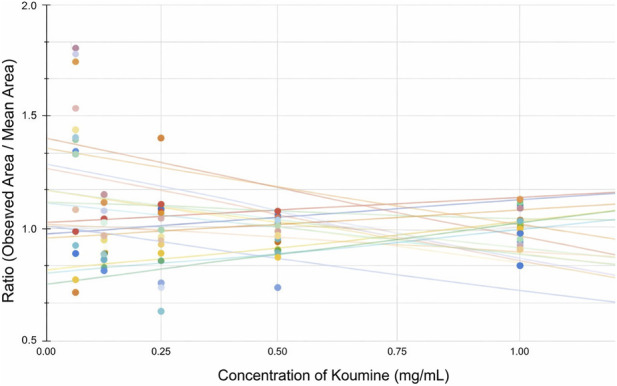
Ratio Plot of observed response to the mean response for each plate as against concentration.

Based on the results acquired and collected, it can be concluded that the leaf contains the least koumine of all the plant’s components, with an average of only 1.273 μg/mg (1.032–4.281 μg/mg). On the other hand, the amount of koumine detected in the stem and root was 2.273 μg/mg (1.130–7.454 μg/mg) and 5.160 μg/mg (1.322–7.561 μg/mg), respectively. Besides that, it can be observed that koumine was either absent or detected in amounts below the LOQ in more than 40% of leaf samples. As a result of the available data, it is safe to conclude that the roots have the highest concentration of koumine, followed by the stem and leaves.

However, comparing the average quantity of koumine present in different parts may be imprecise in light of other parameters, such as geographical origin. In order to enable a precise comparison of the three parts and the differences across provinces, 33 out of the 67 samples were selected, and the average quantity of koumine was calculated. The 33 samples were chosen based on the 11 samples of all three portions of *G. elegans*. For example, samples chosen for the stem, the root, and the leaf were GW037-S, GW051-R and GW052-L because they were from the same specific location, Fujian Province, Pu Tian, Xian You, You Yang, Wu Xing. The other 30 samples selected were GW035-S, GW050-R, GW058-L; GW023-S, GW046-R, GW059-L; GW012-S, GW041-R, GW056-L; GW033-S, GW045-R, GW066-L; GW034-S, GW043-R, GW067-L; GW027-S, GW048-R, GW061-L; from Fujian Province and GW032-S, GW049-R, GW062-L; GW022-S, GW044-R, GW063-L; GW029-S, GW042-R, GW053-L; GW024-S, GW040-R, GW054-L from Guangxi Province.

According to the average concentration of koumine in different parts of *G. elegans* collected in Fujian Province, the root of Fujian Province contains the most koumine of all three parts (Table 6), with the average quantity present being the greatest at 6.655 μg/mg (4.489–9.000 μg/mg), followed by the stem, 2.407 μg/mg (1.211–7.454 μg/mg). Lastly, the leaf contained the least amount, which is 1.552 μg/mg (1.592–4.281 μg/mg). Besides that, the quantity of koumine could not be determined in more than 40% of leaf samples and 25% of stem samples, which was discovered when the amount was less than the LOQ.

The average concentration of koumine in different parts of *G. elegans* from Guangxi province differed significantly in terms of the mean koumine collected from all three parts as well as the overall average quantity obtained (Table 6). The Guangxi province’s roots, comparable to those from Fujian, contained the highest concentration of koumine. However, the average quantity was higher at 2.550 μg/mg (1.322–5.159 μg/mg). This was then followed by the leaf samples that contained an average of 1.581 μg/mg (1.032–3.036 μg/mg) koumine, then lastly, the stem contained the least amount, which is 0.666 μg/mg (1.147–1.518 μg/mg) (Table 6). As shown, the amount of stem is significantly below the LOQ, with 50% of samples falling below it. For the root and leaf, approximately 25% of samples fell below the LOQ. The standard error of the mean of the samples is relatively high. This is because the analysis used a small number of samples; only four samples were chosen for each part of the plant.

## 4 Conclusion

By using the validated HPTLC method, the content of koumine across various parts of *G. elegans* from different geographical areas was successfully differentiated and quantified. The HPTLC fingerprinting method used ethanol: water (7:3) for extraction and development under normal phase silica gel 60 F254 plates, and a mobile phase containing chloroform: methanol: water (30:10:1). Under these conditions, the study detected characteristic bands for each part of *G. elegans*, specifically the stem, leaf, and root. The reference compound, koumine, was also qualitatively detected at *Rf* 0.58. Additionally, a quantitative analysis was conducted to determine the amount of koumine present in each part of *G. elegans*. The study found that the amount of koumine in the stem, root, and leaf samples ranged from 1.130–7.454 μg/mg, 1.322–7.561 μg/mg, and 1.032–4.281 μg/mg, respectively. For the quantification of the Fujian samples, the amounts of koumine found in the stem, root, and leaf are 1.211–7.454 μg/mg, 4.489–9.000 μg/mg, and 1.592–4.281 μg/mg, respectively. The stem, root, and leaf samples from Guangxi yielded the following amounts: 1.147–1.518 μg/mg, 1.322–5.159 μg/mg, and 1.032–3.036 μg/mg, respectively. It can be confidently stated that, based on the samples analysed, the roots of *G. elegans* have a significantly higher concentration of koumine than the stem and leaves, regardless of the province. The HPTLC assay provided us with in-depth insight and a blueprint for the unique fingerprints of samples from various parts of *G. elegans*. HPTLC methods should be used in quantitative manner to prevent the production of excessively diluted botanical ingredients (Gafner et al., 2023). Not only that, *G. elegans is* a highly poisonous plant where misidentification or adulteration can cause lethal public health consequences.

The integration of the iterative factor into the validated HPTLC method can improve the reliability of the calibration process for the quantification of koumine in *G. elegans*. The amount of koumine quantified by this method is comparable to that obtained by the conventional method. Using this method, only one calibration curve is needed for quantifying koumine content on each plate. Hence, a lower sample load is needed on the HPTLC plate. The enhanced method becomes highly suitable for high-throughput routine quality control of *G. elegans*. Although HPTLC inter-plate variability can be influenced by specific analyte chemistry and matrix composition, the successful application of this iterative calibration approach to koumine as well as the previous successful application to kinsenoside in *Anectochilus roxburghii* (Chen et al., 2021), prove its transferability to other botanical matrices. While future applications still require case-by-case validation to justify the unique derivation and matrix effects, this validated protocol does establish a robust framework that can be adapted to other medicinal plants. Ultimately, it can support public health and product quality assurance.

## Data Availability

The raw data supporting the conclusions of this article will be made available by the authors, without undue reservation.
